# Cloning and expression analysis of *SERK1* gene in *Diospyros lotus*

**DOI:** 10.1515/biol-2022-0490

**Published:** 2022-09-26

**Authors:** Ruijin Zhou, Yingying Wang, Xiaona Zhang, Fengqin Jia, Yunli Liu

**Affiliations:** School of Horticulture and Landscape Architecture, Henan Province Engineering Research Centers of Horticultural Plant Research Utilization and Germplasm Enhancement, Henan Institute of Science and Technology, No. 90, East Section of Hualan Avenue, Hongqi District, Xinxiang, Henan 453003, China; School of Horticulture and Landscape Architecture, Henan Institute of Science and Technology, Xinxiang, Henan 453003, China

**Keywords:** *Diospyros lotus*, *SERK*, bioinformatics analysis, gene expression

## Abstract

Somatic embryogenesis receptor-like kinases (*SERKs*), a subfamily of receptor-like kinases, play important roles in response to abiotic stresses in addition to apomictic reproductive development in numerous plant species. The purpose of the present work was to determine if an ortholog of the *SERK* gene is present in the *Diospyros lotus* genome, isolate it and analyze its expression during embryogeny and abiotic stress. An ortholog of the *SERK* gene was isolated from the *D. lotus* genome, and designated as *DlSERK1*. The physical and chemical properties, protein structure, and evolutionary relationship of the *DlSERK*1 protein were analyzed by bioinformatics methods, and the expression of *DlSERK1* gene during embryonic development and under low-temperature, salt, and drought stresses was examined through real-time quantitative PCR analysis. *DlSERK1* contained 1,881 bp open reading frame encoding 626 amino acids, with a molecular mass of 69.18 kDa and pI of 5.34. *DlSERK1* had strong hydrophilic property, signal peptide cleavage sites, and two transmembrane regions, indicating that *DlSERK1* is a secretory protein. The secondary structure of *DlSERK1* was consistent with the tertiary structure, both of which were dominated by random curls and alpha-helices. *DlSERK1* had the typical structure of SERK proteins, and harbored multiple phosphorylation and glycosylation sites. Quantitative analysis showed that *DlSERK1* was expressed during the embryonic development period, and the highest expression level was at 10 days post-flowering. The *DlSERK1* expression level was down-regulated under low-temperature stress and up-regulated under drought and salt stresses. Our study showed that *DlSERK1* was expressed in embryo development and could respond to low-temperature, drought, and salt stresses, which lays a foundation for further research on the function of *SERK1* in the apomixis growth and development of environmental adaptation in *D. lotus.*

## 1 Introduction

Apomictic reproduction in plants is a kind of asexual reproduction in which the megaspore mother cell or nucellar cell directly develops into embryos and then forms seeds without the meiosis and double fertilization of sexual reproduction [1]. Apomictic progenies produced by apomixis inherit the same traits as their female parent without character separation, which speeds up the fixation of heterosis [2,3]. Apomictic reproduction could also be applied to cultivate high-quality rootstocks and establish a stable and efficient somatic embryo regeneration and genetic transformation system *in vitro* [4,5]. Therefore, studying apomictic reproductive characteristics in fruit breeding and production is of great practical value. Apomixis is not only affected by environmental conditions but regulated by multiple genes. At present, many genes related to apomictic reproductive development have been identified, such as *SOMATIC EMBRYOGENESIS RECEPTOR-LIKE KINASE* (*SERK*), *BABY BOOM* (*BBM*), *LEAFY COTYLEDON 2* (*LEC2*), *AGAMOUS-LIKE 15* (*AGL15*), *WUSCHEL* (*WUS*), and *FUSCA3*. *SERK* belongs to the leucine-rich repeat sequence receptor-like kinase (LRR-RLK) family [6], widely distributed in plants. Schmidt et al. [7] discovered the first *SERK* gene (*DcSERK*) in the hypocotyl of carrot. Subsequently, *SERK* genes were isolated from *Arabidopsis thaliana* [8], rice (*Oryza sativa*) [9], maize (*Zea mays*) [10], barley (*Hordeum vulgare*) [11], wheat (*Triticum aestivum*) [12], grape (*Vitis vinifera*) [13], and apple (*Malus hupehensis*) [14], and were shown to play an important role in somatic embryogenesis. In general, the *SERK* gene consists of 11 exons and 10 introns, and the exons almost correspond to the functional domain of the coding protein, including the leucine-zipper (ZIP), leucine-rich repeat sequence (LRR), proline-rich (SPP), transmembrane (TM), and kinase domains, as well as the N- and C-terminal regions [15,16], which participate in important biological functions [17].

The involvement of the *SERK* gene in sporophyte development has been reported in *A. thaliana*. For example, *AtSERK1* and *AtSERK2* are expressed in both vegetative and reproductive tissues of *A. thaliana*, but their expression levels are relatively high in flowers and fruit pods, indicating that they may be involved in controlling sporophyte differentiation and thus affecting the development of male gametophytes [18]. At the same time, SERKs may function as coreceptors of *GS01/2* to transduce the *TWS1* signal and ultimately regulate embryonic cuticle integrity [19]. SERKs play a critical role in regulating zygotic embryo development through controlling the division patterns of vascular precursors and ground tissue stem cells. [20] *AtSERK1* is only expressed at the heart-stage during embryo development, which is similar to the expression patterns of *DgSERK* and *DcSERK* [8,21]. In addition, *SERK*s (*SERK1* and *SERK2*) are also involved in microspore embryogenesis in *Brassica napus* L., and the expression level of *BnSERK1* is significantly up-regulated within 1–5 days after microspore-derived embryogenesis, whereas the *BnSERK2* expression is increased throughout microspore-derived embryogenesis [22]. It has been reported that expression of genes of the *SERK* family in maize are associated with embryogenesis induction [10]. Therefore, *SERK* genes play an important role in microspore development and reproductive development. However, SERKs also show differentiated functions, they play crucial roles in many biological processes such as brassinosteroids (BR) signaling, anther development, stomatal patterning, floral abscission, immune responses, hormone signal transduction, disease defense, and abiotic stress response [20,23–32]. For instance, *AtSERK3-5* are involved in the regulation of BR signaling, affecting the growth and development of *A. thaliana* [29,30]. In rice, although *OsSERK1* and *OsSERK2* are expressed in all tissues, *OsSERK1* is mainly expressed in flowers and stems, while *OsSERK2* is mainly expressed in leaves. The expression of *OsSERK1-2* can also be activated by pathogens, host dead cells, defense signaling molecules (such as salicylic acid), and other stress signals, modulating the immune signaling pathways [9,31]. Under salt stress, the expression of *HvSERK1* and *HvSERK3* in barley leaves was up-regulated at 12 h, and *HvSERK1* remained at a high level until 24 h, while the *HvSERK3* expression was decreased to a normal level at 24 h, suggesting that *HvSERK1* and *HvSERK3* may be involved in the salt tolerance pathway of barley leaves [11].

*Diospyros lotus* is a dioecious plant. We found that some females of *D. lotus* have apomixes characteristics [33], this has great application potential for *D. lotus* breeding research. But the type of apomixes in *D. lotus* is not clear enough, in particular, the regulatory mechanism of apomixes is unclear. *SERK* genes have been isolated and identified in many plants, but there are less reports on *SERK* genes in *D. lotus*. Lijie et al. [14] found that the *SERK1* gene was highly expressed in the ovary at the bud stage of *M. hupehensis* var. *pingyiensis* Jiang, while *CitSERK1* and *CitSERK1-LIKE* genes were highly expressed in the somatic embryo induction process of citrus [34,35]. In view of the important role of *SERK*s and their homologous genes in sporophyte development, reproductive development, and related resistance in higher plants, we cloned *SERK* homologous genes from *D. lotus*, and analyzed their expression patterns under somatic embryogenesis, low temperature, drought, and salt stress by reverse transcription quantitative PCR (RT-qPCR). At the same time, the results obtained from bioinformatics analysis lay a foundation for further research on the function of *SERK* genes and provide theoretical support for the study of the molecular mechanism of *SERK* genes in the growth and development process and environmental adaptation of *D. lotus*.

## 2 Materials and methods

### 2.1 Plant material

The samples were collected from the perennial *D. lotus* L. tree grown at the test base of Henan Institute of Science and Technology.

### 2.2 Sampling during apomictic reproductive development

When *D. lotus* flowers were not open, the petals changed from green to yellow, the petals surrounding the stigmas were gently removed. Polyvinyl acetate emulsion adhesive was applied evenly on the stigma to completely wrap the stigma [33]. To ensure that pollen did not enter the stigma, each stigma was treated three times at an interval of 12–24 h. The samples were collected 1 day after treatment (as the control). Afterward, samples were taken once every 3 days until white embryos were formed in the fruit of *D. lotus*. The samples were immediately frozen in liquid nitrogen, brought back to the laboratory, and stored at −80°C.

### 2.3 Abiotic stress treatment

Sufficient seeds with full grain and uniform size were sown in a nutrient bowl. The treatment was performed on seedlings with three or four leaves. Low-temperature treatment: *D. lotus* seedlings with the same growth status were placed in an incubator at 4°C, under continuous light supply and normal watering. Drought treatment: root irrigation was applied to robust *D. lotus* seedlings with same sizes with 20% PEG 6000 until saturation. Salt treatment: root irrigation was applied to healthy *D. lotus* seedlings with consistent growth with 250 mM NaCl solution until saturation. The third leaf from bottom to top was collected at 0 h (CK), 6 h, 12 h, 1, 3, 5, and 7 days after abiotic treatment, frozen immediately in liquid nitrogen, and stored at −80°C until further use. Three biological replicates were conducted for each treatment. The relative electrical conductivity (REC) of the leaf at each time point was measured by a DDS-307 electrical conductivity meter, and the electrode constant was 0.990. According to the method of Bolat with some modifications [36], the conductivity of sample solution was *R*_1_, and that of deionized water partake solution was *R*_A_ before boiling. After boiling and cooling, the conductivity of sample solution was *R*_2_, and that of deionized water counterpart solution was *R*_B_. Then, REC (%) = (*R*_1_ – *R*_A_)/(*R*_2_ – *R*_B_) × 100%. Three biological replicates were selected at each time point of each treatment.

Total RNA was extracted using the OminiPlant RNA Kit (DNaseI, CW2598S; Kangwei Century Biotechnology Co., Ltd) following the manufacturer’s instructions. cDNA was synthesized by the RevertAid First Strand cDNA Synthesis Kit (#K1622; Thermo Scientific) and stored at −20°C for later use.

### 2.4 Screening and cloning of *SERK* in *D. lotus*

The *SERK* gene sequence was screened from the genome database of *Diospyros oleifera* “Youshi,” and specific primers to amplify the full length of the *SERK* gene were designed (upstream primer: 5′−ATGGAAAGATTGGTGTTGGTGA−3′; downstream primer: 5′−TCACCTGGGCCCTGATAACT−3′). PCR amplification was carried out using cDNA from the leaves of the explants as the template by the homologous cloning method. The PCR reaction system was 20 μL, containing 1 μL of cDNA, 0.5 μL of each primer (10 μM), 10 μL of 2× Es TaqMster Mix (Dye), and 8 μL of ddH_2_O. The PCR reaction conditions were pre-denaturation at 94°C for 4 min; 94°C for 30 s, 56°C for 30 s, 72°C for 90 s, 35 cycles; extension at 72°C for 5 min. The target fragment was separated by 1.0% agarose gel electrophoresis and recovered by the Agarose gel DNA Recovery Kit. After the pMD18-T vector (TaKaRa) was connected, the plasmid was transformed into *Escherichia coli* DH5α strain by heat shock (laboratory preservation), and positive clones were identified with sequencing conducted by Sangong Biotech Co., Ltd (Shanghai, China). According to the sequencing results, the recovered fragment was confirmed as the *SERK* gene based on multiple characteristics of protein and gene structure and was named *DlSERK1*.

### 2.5 Bioinformatics analysis of *DlSERK1*

The online software BLASTP was used for gene sequence search and analysis (https://blast.ncbi.nlm.nih.gov/Blast.cgi). The full-length open reading frame (ORF) was predicted by the online tool ORF-finder (http://www.ncbi.nlm.nih.gov/gorf/gorf.html). The physicochemical properties and hydrophilicity of the encoded *DlSERK1* protein were predicted by ProtParam (http://web.expasy.org/protparam/) and ExPASy (http://web.expasy.org/protscale/). SOPMA (https://npsa-prabi.ibcp.fr/cgi-bin/npsa_automat.pl?page=npsa_sopma.html) and SWISS-MODEL online tools were used to analyze the secondary structure and three-dimensional structure composition of the *DlSERK1* protein. TM helices were analyzed using the TMHMM server (http://www.cbs.dtu.dk/services/TMHMM/). The subcellular localization of *DlSERK* was predicted using Plant-mPLoc (http://www.csbio.sjtu.edu.cn/bioinf/plant-multi/) and PredictProtein (https://predictprotein.org/). Signal peptides were predicted by SignalP v5.0. The Netphos v3.1 server, NetOGlyc v4.0, and NetNGlyc v1.0 software were used to predict phosphorylation and glycosylation sites in *DlSERK*. The MotifScan program (http://myhits.isb-sib.ch/cgi-bin/motif_scan) was used to analyze the conserved motifs. Sequence alignment was performed using DNAMAN v9.0. Phylogenetic trees were prepared with MEGA v5.0 software using the neighbor-joining (NJ) method. The 2,000-bp sequence upstream of *DlSERK1* was regarded as the promoter region and extracted from the *D. lotus* genome database (http://persimmon.kazusa.or.jp/blast.html) by TBtools and submitted to the Plant CARE database for identifying the *cis*-acting elements.

### 2.6 RT-qPCR

The fruits which were capped by white latex for 1, 4, 7, 13, 16, 19, and 22 days and leaves treated with low temperature, drought, and salt stress were used as test materials. RNA was extracted, reverse transcribed to synthesize cDNA, and real-time quantitative PCR was performed. The RT-qPCR was performed on a CFX96 instrument (Bio-Rad), and the PCR reaction was prepared according to the instruction of the SYBR Green qPCR Kit (TaKaRa). Primer sequences were: SERKrt1F, TGCCATCTGAACCACCACTC and SERKrt1R, ACGGCTGTAATGACGTGTGT. The 10-μL PCR reaction system contained 5.0 μL of SYBR Premix Ex Taq II enzyme, 0.7 μL of cDNA, 0.4 μL of each primer (10 μM), and 3.5 μL of ddH_2_O. The amplification parameters were as follows: 95°C pre-denaturation for 30 s, 1 cycle; 95°C denaturation for 5 s, 56°C renaturation for 30 s, 40 cycles. The comparative threshold 2^−ΔΔCT^ method was applied to quantify the relative expression of target genes [37]. Four independent assays were carried out.

## 3 Results

### 3.1 Cloning of the *SERK1* gene in *D. lotus*

**Figure 1:**
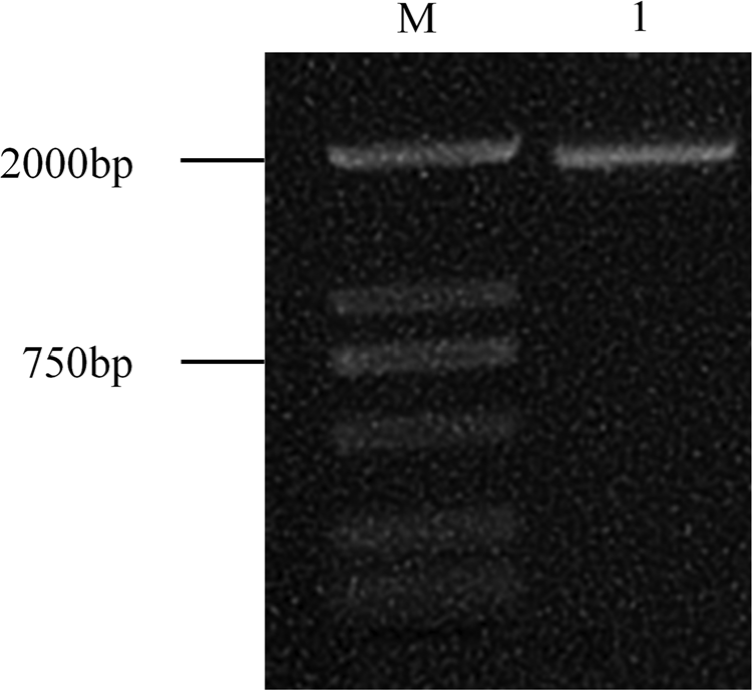
Amplification of *DlSERK1*. M: DL2000 DNA marker; 1: amplification of full-length cDNA of *DlSERK1*.

**Figure 2:**
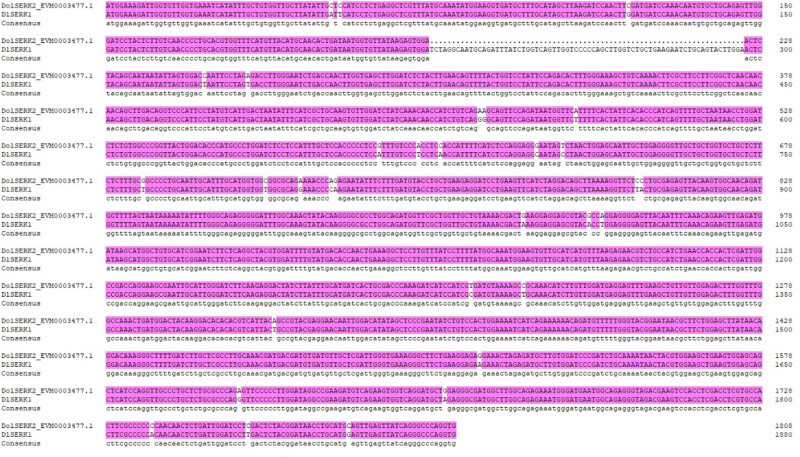
Nucleotide sequences of the *SERK* gene in *D. oleifera* Cheng and *D. lotus.*

cDNA of *D. lotus* leaves was used as the template for PCR amplification. After cloning and sequencing, the ORF length of *DlSERK1* sequence was 1,881 bp (Figure 1). Comparison of nucleotide sequence length showed that the *DlSERK1* gene was 72 bp longer than *SERK* of *D. oleifera*, and these two sequences length shared 96.17% similarity (Figure 2). Moreover, compared with the homologous *SERK* sequences in other plants, this fragment showed 100% similarity with tetraploid hybrid offspring of *Malus*, 100% similarity with *PpSERK2*, and 98.90% similarity with *SERK1* of *D. officinale*, indicating that the full-length cDNA sequence of *DlSERK1* was cloned.

### 3.2 Bioinformatics analysis of *DlSERK1*

#### 3.2.1 Homology of *DlSERK1* protein

Through BLASTP searches against the NCBI database, the protein sequence encoded by *DlSERK1* showed high similarity with *SERKs* of other species. The similarities between the *DlSERK1* protein with *SERKs* of *Sesamum indicum* (XP_011074139.1), *Populus euphratica* (XP_011002514.1), *V. vinifera* (XP_002270847.1), *Carica papaya* (ABS32233.1), and *Solanum lycopersicum* (NP_001233866.1) were 93.25, 92.27, 91.94, 91.56, and 89.94%, respectively, indicating that the cloned *DlSERK1* gene was homologous with *SERK*s.

#### 3.2.2 Physicochemical properties of *DlSERK1*

The ORF of *DlSERK1* was 1,881 bp in length and encoded a protein of 626 amino acids that had a calculated molecular mass of 69.18 kDa and a predicted pI of 5.34. The molecular formula of the *DlSERK1* protein was C_3109_H_4893_N_839_O_907_S_20_, its lipid solubility index was 101.37, and its instability coefficient was less than 40 (39.52), belonging to the stable protein. Among the 20 amino acid residues in *DlSERK1*, leucine was the most (15.5%), followed by glycine (7.8%), while cysteine (1.3%) was the least. The total number of negatively charged residues (Asp + Glu) was 74 and that of positively charged residues (Arg + Lys) was 56. The total average hydrophilic coefficient of *DlSERK1* was –0.098, suggesting that *DlSERK1* is a hydrophilic protein.

#### 3.2.3 Hydrophilicity of *DlSERK1* protein

**Figure 3:**
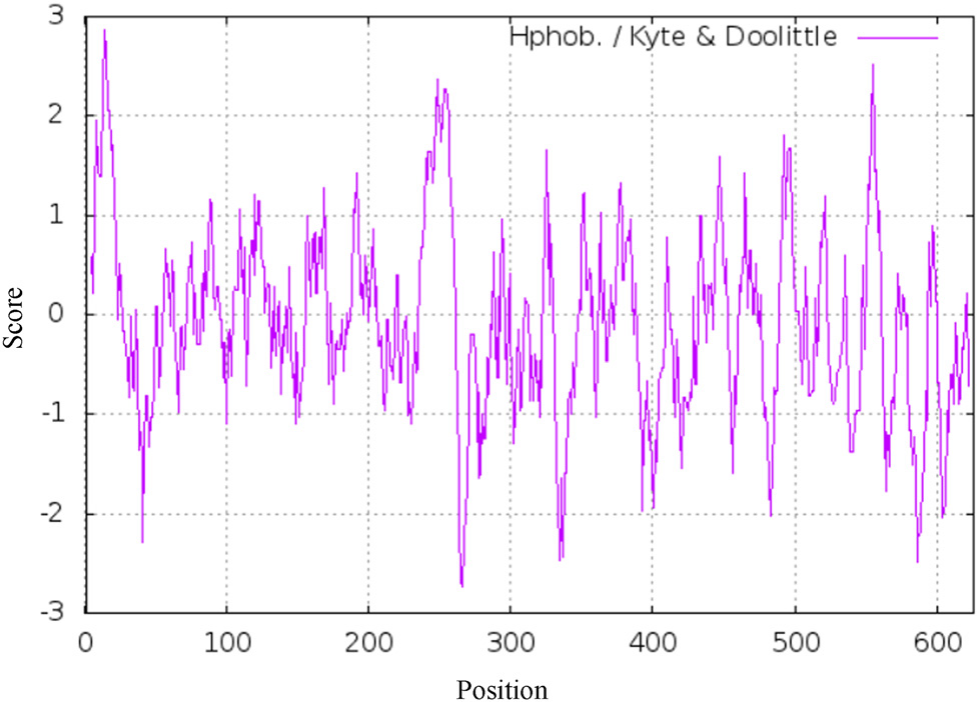
Predicted hydrophilic sites of *DlSERK1*.

Analysis by ExPASy software showed that there were several strong hydrophilic regions in the N-terminal, C-terminal, and intermediate regions of *DlSERK1* (Figure 3). The site with the strongest hydrophilicity was found at amino acid residue 266 (Lys), with a value of −2.733. The most hydrophobic site was found to be residue 14 (Leu), with a value of 2.867.

#### 3.2.4 Secondary and tertiary structures of *DlSERK1* protein

**Figure 4:**
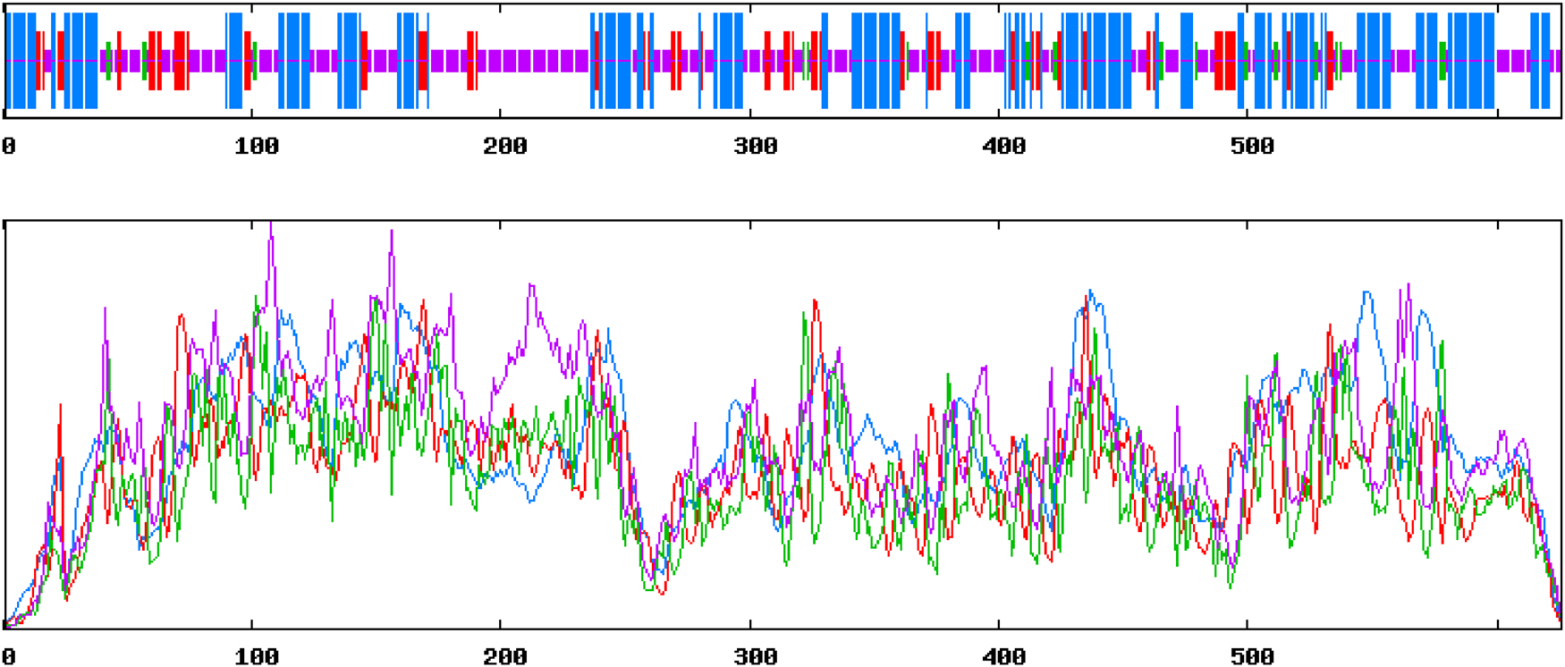
Predicted secondary structure of *DlSERK1*.

**Figure 5:**
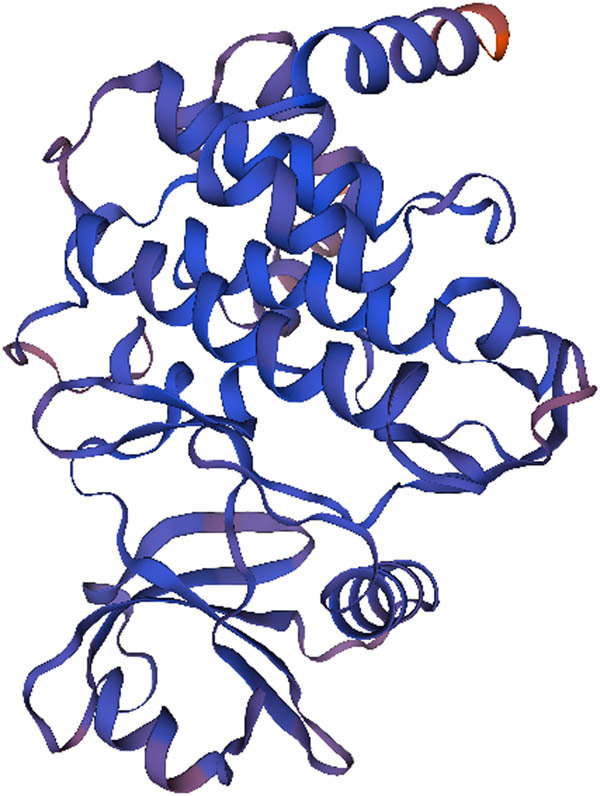
Predicted tertiary structure of *DlSERK1*.

The secondary structure prediction of *DlSERK1* (Figure 4) revealed that it was mainly composed of 39.30% alpha-helices (blue line), 4.31% beta-turns (green line), 42.65% random coils (purple line), and 13.74% extended strands (red line). Through homology modeling, it was found that the tertiary structure of *DlSERK1* (Figure 5) had the most similarity with the 3tl8.2.A model, and the tertiary structure was dominated by random coils and alpha-helices, which is consistent with the prediction results of secondary structure.

#### 3.2.5 Subcellular localization, TM structure, and signal peptide prediction of *DlSERK1*

**Figure 6:**
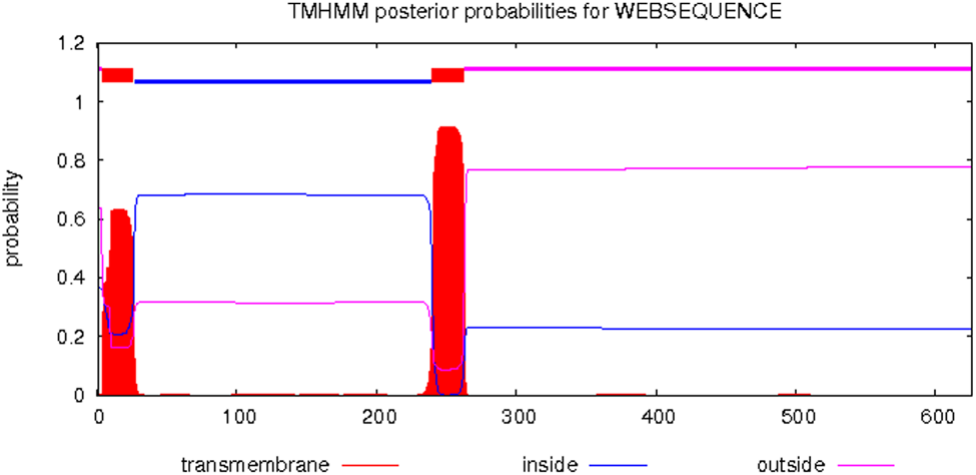
Predicted TM domain of *DlSERK1*.

*DlSERK1* was predicted to be localized to the cell membrane considering the results from both plant-MPLOC and Predict Protein. TM prediction results (Figure 6) showed that *DlSERK1* contained two TM domains, i.e., TM helices at amino acid residues 4–26 and 240–262, respectively. Therefore, *DlSERK1* was presumed to be a TM protein. The *DlSERK1* protein may contain a signal peptide (Sec/SPI), and the cleavage site was between residues 26 and 27, with a probability of 0.98 (CS > 0.5), indicating that the protein belongs to the secretory protein.

#### 3.2.6 Prediction of phosphorylation and glycosylation sites of *DlSERK1* protein

**Figure 7:**
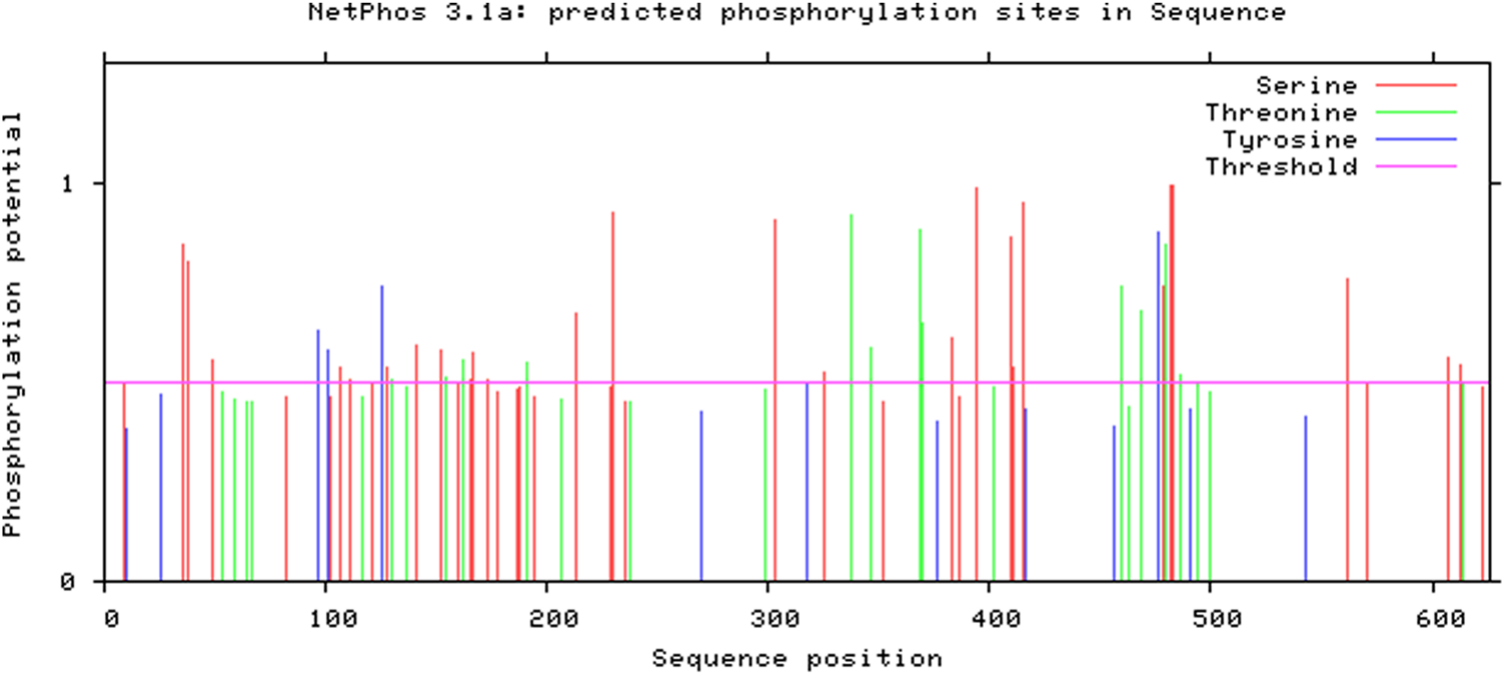
Predicted phosphorylation sites of *DlSERK1*.

The phosphorylation sites of *DlSERK1* were predicted by the NetPhos v3.1 server (Figure 7). The results showed that there were 43 phosphorylation sites in *DlSERK1*, including 27 serine (Ser) sites, 12 threonine (Thr) sites, and four tyrosine (Tyr) sites. NetOGlyc v4.0 and NetOGlyc v1.0 were used to predict the O-terminal and N-terminal glycosylation sites in *DlSERK1*. Three O-terminal glycosylation sites were located at residues 394 and 607, respectively, and five N-terminal glycosylation sites were located at residues 115, 150, 163, 184, and 381.

#### 3.2.7 Conserved motif prediction of *DlSERK1* protein

Analysis based on MotifScan showed that *DlSERK1* contained several typical conserved domains of *SERK* proteins, including an N-terminal region LRRNT_2 (between residues 26 and 66), a leucine zipper (between residues 33 and 54), four leucine repeats LRR_1 (between residues 94 and 116, 118 and 140, 142 and 164, and 166 and 189), a proline-rich domain with the Ser-Pro-Pro (SPP) motif, a TM domain (between residues 204 and 231), a TM domain (between residues 240 and 262), and a protein kinase active domain comprising 11 sub-domains (between residues 302 and 589). Additionally, a protein kinase ATP-binding site (between residues 308 and 330) and a serine/threonine kinase activation site (between residues 425 and 437) were observed.

#### 3.2.8 Multiple alignment of amino acid sequences and construction of phylogenetic tree

**Figure 8:**
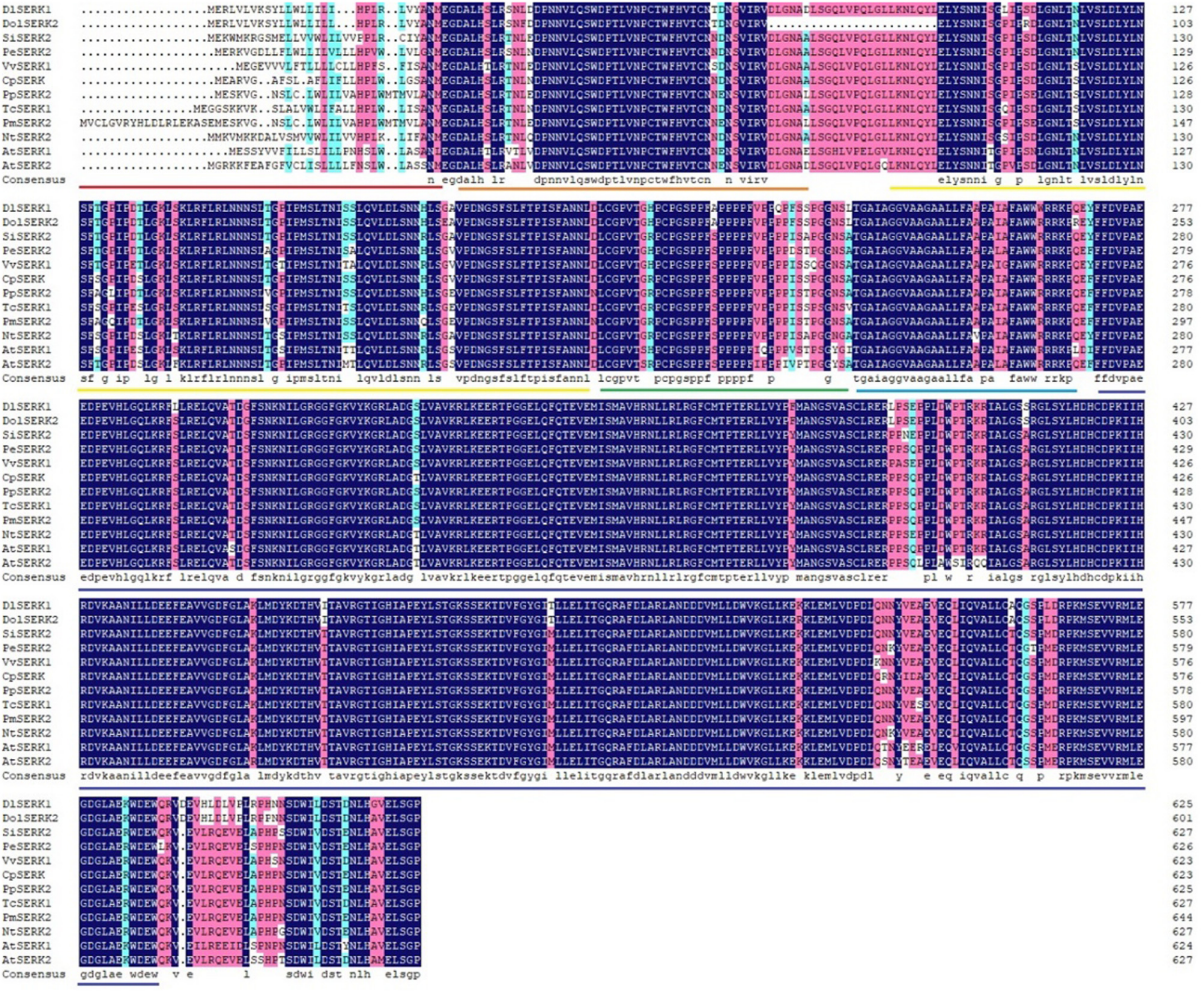
Multiple sequence alignment of amino acid sequences of *DlSERK1* and *SERKs* in other plants. Conserved sequence characteristics of *SERK* are indicated with colored underlines (red, signal peptide; orange, leucine zipper; yellow, leucine repeat sequence; green, serine-proline-rich domain; blue, TM domain; and purple, kinase domain).

The protein sequences of *SERKs* from other plant species were downloaded from NCBI, including *D. oleifera* Cheng (AKN89445.1), *S. indicum* (XP_011074139.1), *P. euphratica* (XP_011002514.1), *V. vinifera* (XP_002270847.1), *C. papaya* (ABS32233.1), *Prunus persica* (XP_007201734.1), *Theobroma caca* (XP_007020220.1), *Prunus mume* (XP_008245841.1), *Nicotiana tabacum* (XP_016455663.1), and *A. thaliana* (ACN59271.1 and CAF33246.1). Multiple sequence alignment was performed by DNAMAN. The results showed that the similarity among these sequences was 90.81%. Among them, *DlSERK1* had the highest similarity with *D. oleifera SERK2* (94.47%), followed by *SiSERK2* (93.25%). Analysis results showed that these sequences had typical structural and functional domains of *SERK* proteins (Figure 8). This confirms that *DlSERK1* is a member of the *SERK* family and belongs to *SERK/LRR-RLK* genes.

**Figure 9:**
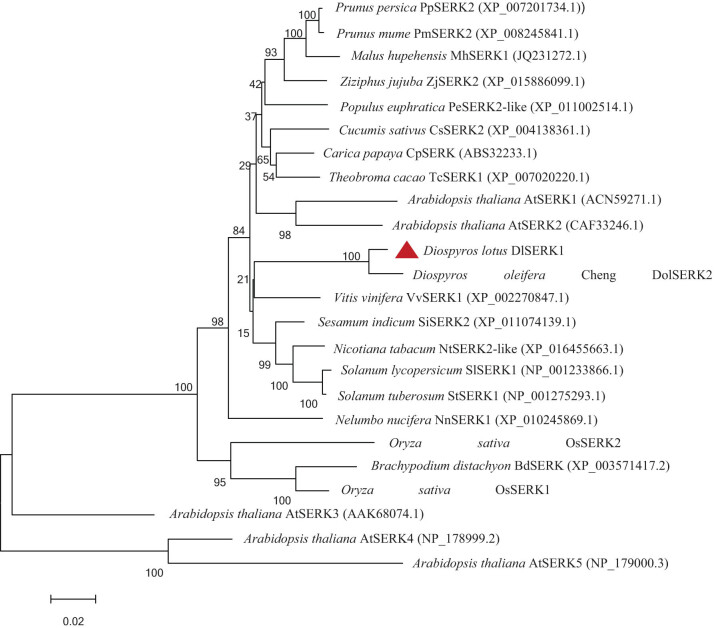
Phylogenetic tree of *DlSERK1* and *SERKs* in other plants.

To explore the evolutionary relationship between *DlSERK1* and *SERKs* from other species, a phylogenetic tree was constructed by MEGA v5.0 based on the NJ method (Figure 9). It was found that *DlSERK1* was closest to *DolSERK2*; the two were clustered in a small clade with *VvSERK1* first, and then clustered with *SiSERK2*, *NtSERK*, *SlSERK1*, and *SERKs* of other dicotyledons. *SERK* sequences were clustered in different clades of monocotyledonous plants including *Brachypodium distachyon* and rice, and were relatively distant from each other. The results of the phylogenetic tree were consistent with the homology comparison results. Among dicotyledons, *SERKs* of peach, plum, and apple of Rosaceae belonged to one group, and those of tobacco, tomato, and potato of Solanaceae belonged to another group. While *AtSERK1* and *AtSERK2* were clustered to one branch, *AtSERK3*, *AtSERK4*, and *AtSERK5* were in separate clades, indicating that different *SERK* proteins in the same plant species can also be divergent.

#### 3.2.9 Analysis of regulatory elements in the *DlSERK1* promoter

The 2,000 bp nucleotide sequence upstream of the *DlSERK1* gene was analyzed by Plant CARE to obtain the regulatory elements (Table 1). The prediction results showed that this sequence contained not only a large number of the core sequence (TATA-box) in the promoter, the CAAT-box in the promoter and enhancer regions, and other basic promoter elements across eukaryotes, but also included nine light-responsive elements (G-box, GATA-motif, TCT-motif, and AE-box), 12 hormone response-related *cis*-elements (TGACG-motif, CGTCA-motif, ABRE, and P-box), two stress response-related *cis*-elements (ARE and MBS), and two responsive elements involved in the regulation of corn protein metabolism (O2-site).

**Table 1:** *Cis*-acting elements of the *DlSERK1* gene

Type of *cis*-acting element	Associated element	Number	Function of response
Light response-related element	G-box	Five	Light-responsive element
	GATA-motif	Two	Light-responsive element
	TCT-motif	One	Light-responsive element
	AE-box	One	Light-responsive element
Hormone response-related *cis*-element	TGACG-motif	Four	MeJA responsiveness
	CGTCA-motif	Four	MeJA responsiveness
	ABRE	Three	Abscisic acid responsiveness
	P-box	One	Gibberellin-responsive element
Stress response-related *cis*-element	ARE	One	Anaerobic-induction element
	MBS	One	MYB binding site involved in drought inducibility
Growth-related *cis*-element	O2-site	Two	Zein metabolism regulation

### 3.3 Changes of REC of leaves under abiotic stress

**Figure 10:**
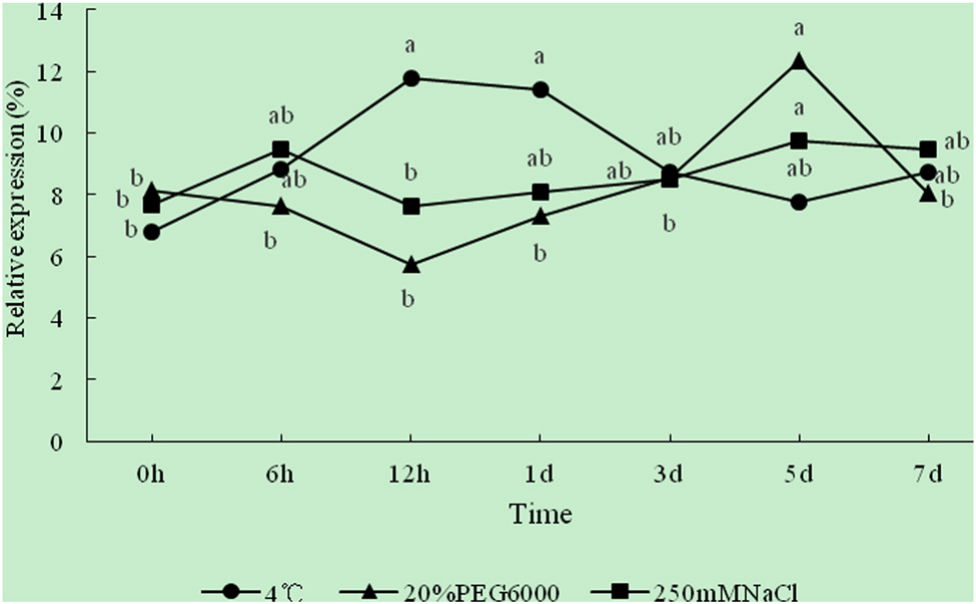
Relationship between treatment time and REC under different stress treatments. Different lowercase letters indicate significant differences at *P* < 0.05.

In order to calibrate the degree of cell damage under low temperature, drought, and salt stress at different times, and to determine the rationality of the sampling time of gene expression in the later period, we measured the REC. With the extension of the treatment time, the REC of leaves increased in varying degrees compared with CK (0 h), indicating that the cell membrane was damaged to varying degrees (Figure 10). The REC of leaves increased linearly within 12 h under 4°C and peaked (11.76%) at 12 h, which was increased by 4.96% compared with CK. Although a downward trend was observed after 12 h, the REC was always higher than that of CK. The REC showed a gradual downward trend within 12 h of drought treatment with 20% PEG 6000. At 12 h, the REC of leaves was the lowest (5.74%); it then rose until the maximum value appeared at 5 days (12.32%), which was increased by 4.17% compared to that of CK and 2.15 times relative to that at 12 h. Within 7 days of 250 mM NaCl treatment, the REC first showed an upward trend within 6 h, then decreased to the CK level at 12 h, and then gradually increased. At 5 days, the REC reached a maximum value (9.74%), which was increased by 2.08% compared to CK. The results showed that low-temperature, drought, and salt treatment increased the REC of seedling leaves, and the membrane was damaged under abiotic stress. The damage intensity of the cell membrane under different stresses was in an order of 4°C > 20% PEG 6000 > 250 mM NaCl.

### 3.4 Expression analysis of *DlSERK1*

**Figure 11:**
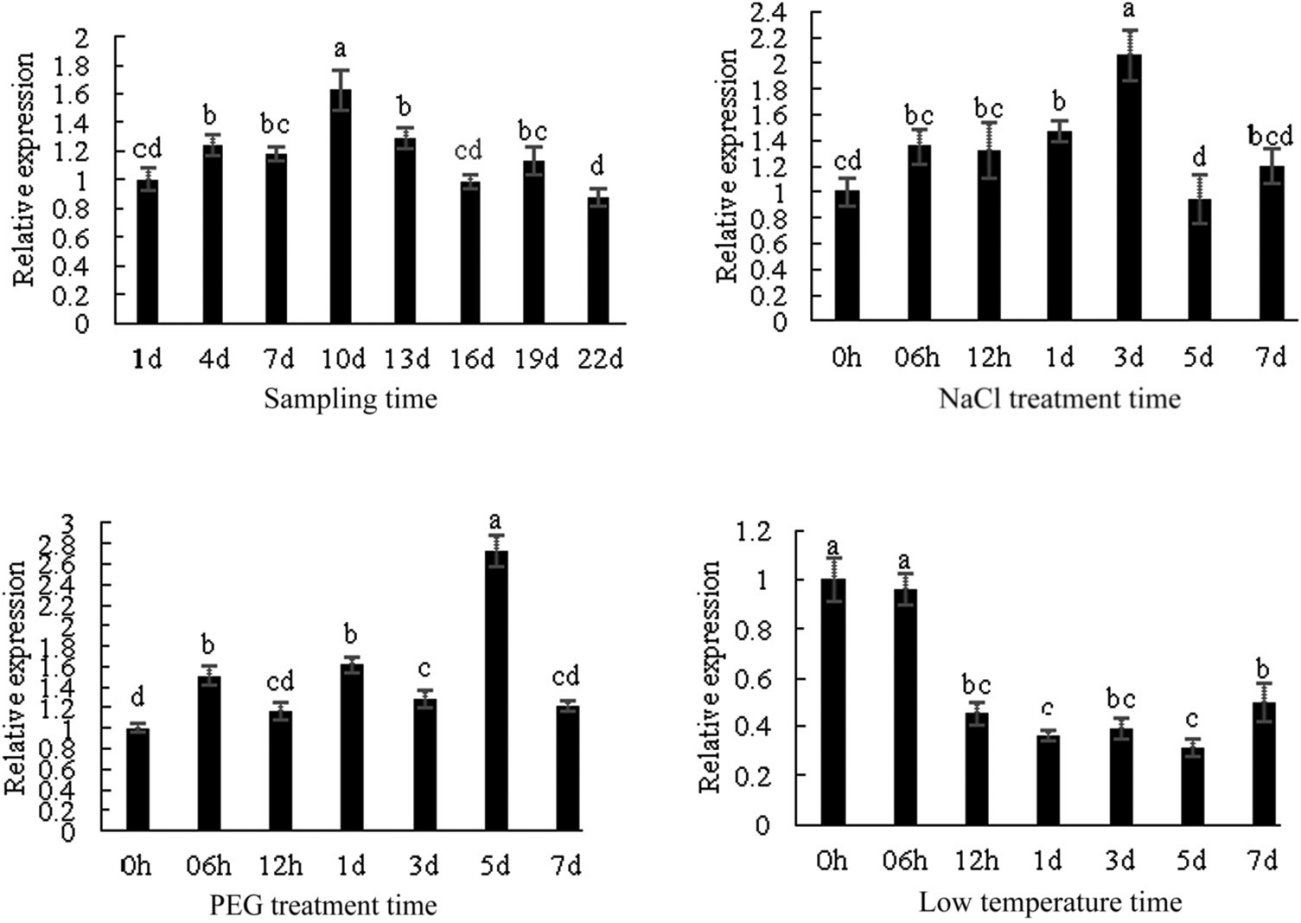
*DlSERK1* gene expression analysis during embryogenesis and abiotic stress.

The expression of *DlSERK1* during the ovaries of different flower development stages in *D. lotus* was analyzed by RT-qPCR (Figure 11). The results showed that *DlSERK1* was expressed during the embryonic development period. At 1–3 days post-treatment (DPT), some flowers bloomed, roughly in bud stage. At 4–7 DPT, most flowers bloomed, which was the full flowering stage. About a week after flowering, the stigma turned brown, and the petals gradually withered, which was the late flowering stage. The highest *DlSERK1* expression in ovary was observed at 10 days of flowering, which was 1.63 times higher than that at Day 1 of flowering. It was speculated that *DlSERK1* may promote the development of somatic embryos.

The expression patterns of *DlSERK1* under various abiotic stresses (4°C, PEG, and NaCl) were studied. *DlSERK1* was expressed in leaves at low temperature for 7 days, and the expression was almost unchanged after 6 h of treatment. The *DlSERK1* expression decreased rapidly after 12 h of treatment, which was less than half of the CK, and remained at a low level until 7 DPT. Under PEG treatment, the expression of *DlSERK1* was up-regulated in leaves, and the peak expression appeared at 5 DPT, which was about 2.73 times that of the CK. Under the condition of high-salt treatment, the expression of *DlSERK1* in leaves increased first and then decreased. The expression increased significantly within 3 days of treatment, and peaked at 3 DPT, which was about 1.9 times that of CK. However, at 5 DPT, the *DlSERK1* expression decreased to the lowest level, and then returned to the normal level at 7 DPT.

## 4 Discussion

In this study, the full-length cDNA of *DlSERK1* (1,881 bp), encoding 626 amino acids, was cloned from the *D. lotus* leaf. Bioinformatics analysis showed that the *DlSERK* protein encoded by this gene had high similarities with *SERK* proteins of other plants. *DlSERK* also contained common conserved structural domains of *SERK* proteins, including a signal peptide, leucine zipper structure, proline-rich structure, TM structure, leucine-repeat sequence, and intracellular protein kinase activity domain, which were the typical structural characteristics of the *SERK* protein family. Therefore, it was speculated that *DlSERK* was a member of the *SERK* protein family and was thus named *DlSERK1*. *DlSERK1* had high similarities to other plant *SERK* proteins in amino acid composition, molecular weight, and pI. For example, *DlSERK1*, *AtSERK1*, *PpSERK2*, *MtSERK1*, *OsSERK1*, and *CitSERK1* proteins were composed of 626, 625, 626, 627, 628, and 621 amino acids, respectively; their molecular weights were 69.18, 69, 68.99, 69.125, 69.5, and 68.4 kDa, respectively; and their pIs were 5.34, 5.25, 5.38, 5.56, 5.98, and 5.48, respectively [9,18,34,38]. The results showed that *SERK* proteins of different plants had similar primary structure and physicochemical properties, thus it was speculated that they play similar roles in the process of plant growth and development. The secondary and tertiary structures were consistent with the prediction results, which revealed mainly random coils and alpha-helices. There was a cleavage site of signal peptide between residues 26 and 27. It was speculated that *DlSERK1* is a secretory protein with a TM structure located on the cell membrane, and harbors multiple phosphorylation and glycosylation sites, which is consistent with the function of *SERK* proteins in signal transduction. *DlSERK1* had the highest similarity with *DolSERK2*, was close to *SERKs* of dicotyledonous plants such as sesame, tobacco, and tomato, and far from *SERKs* of monocotyledonous plants such as rice and *B. distachyon.*

The expression level of *DlSERK1* was the highest at 10 days after flowering, which was the late flowering stage of *D. lotus*. The fruit began to expand about half a month after flowering, and the *DlSERK1* expression returned to the normal level. According to the *DlSERK1* expression at different stages of ovary development, it can be inferred that *DlSERK1* may play a certain regulatory role in embryogenesis in *D. lotus*. This is similar to the results of *D. officinale* [39], citrus [34], and apple [14]. *DoSERK2* was widely expressed at all stages of protocorm development, and the expression was the highest at the stage of seed embryo activation. The expression of *DoSERK2* was low and showed little difference from protocorm formation to the seedling stage. The expression signal of *CitSERK1* could be detected in flowers and fruits at 30 and 60 days after flowering, but not in fruit at 180 days after flowering. The expression levels of *MhSERK1* and *MhdSERK1* were the highest in the ovary of *M. hupehensis* and in the ovary of the hybrid progeny, respectively. The expression of *SERK1* was the highest in the ovary of *M. hupehensis* at the bud stage, while the expression was the highest in the ovary of hybrid progeny at the flowering stage, which may be the main reason for the decline in apomixis ability of hybrid progeny.

A large number of studies have shown that *SERK* proteins play an important role in the regulation of abiotic stress. Ma et al. found that *SERK1* could be induced by low-temperature treatment at 4°C for 24 h in non-embryogenic callus of pineapple [40]. Xuekai et al. found that low-temperature accumulation could induce the *PsSERK2* expression in *Paeonia suffruticosa* “Luhehong,” and *PsSERK2* plays a positive regulatory role in sleep release [41]. In this study, we found that the expression level of *DlSERK1* decreased significantly with the increase of low-temperature treatment time (low temperature for 12 h) by RT-qPCR technique. *DlSERK1* plays a negative regulatory role in low temperature stress. Many gene families play different roles in different plants, such as the MYB gene family has positive and negative regulatory functions [42,43]. Most of the MYBs involved in the control of flavonoid biosynthesis are positive regulators that enhance the expression of the structural flavonoid pathway genes [2]. However, repressors have also been characterized, such as *FaMYB1* in strawberry (*Fragaria x ananassa* Duch.) and *VvMYB4* in the berries of grapevine [44,45].The expression level of *DlSERK1* was up-regulated at 5 DPT during drought stress, which was similar to that of *BnaA07g23390D* and *BnaA07g29610D* in *B. napus* under drought treatment, with the *BnaA07g23390D* expression being up-regulated at 1 h of treatment and the *BnaA07g29610D* expression being up-regulated at 0.5 h [46]. The expression of *DlSERK1* was up-regulated after salt treatment, similar to *GmSERK16*, *HvSERK1*, *HvSERK3*, *MdSERK4*, *MdSERK10*, *BnaC01g43240D*, and *BnaCnng07810D* [32,47–49]. The results indicate that *DlSERK1* can respond to low-temperature, drought, and salt stress signals. Plant CARE analysis showed that *DlSERK1* may be involved in the light-response process, response to the signal regulation of hormones such as MeJA and abscisic acid, and protein metabolism. This indicates that the regulation of gene expression is extremely complex, and *DlSERK1* may have multiple functions and participate in various biological processes.

## 5 Conclusion

In summary, the cloning of *SERK* has laid a foundation for studying the role of this gene in the development of embryogenesis in *D. lotus*, and it is of great significance for the in-depth study of the molecular mechanism of embryonic development and the function of environmental adaptability in *D. lotus*.
